# Draft Genome Sequences of Four Microcystis aeruginosa Strains (NIES-3787, NIES-3804, NIES-3806, and NIES-3807) Isolated from Lake Kasumigaura, Japan

**DOI:** 10.1128/MRA.00052-20

**Published:** 2020-04-02

**Authors:** Haruyo Yamaguchi, Shigekatsu Suzuki, Masanobu Kawachi

**Affiliations:** a Center for Environmental Biology and Ecosystem Studies, National Institute for Environmental Studies, Ibaraki, Japan; University of Southern California

## Abstract

Microcystis aeruginosa is a bloom-forming cyanobacterium found in freshwater environments. The draft genomes of the *M. aeruginosa* strains NIES-3787, NIES-3804, NIES-3806, and NIES-3807, which were isolated from Lake Kasumigaura, Japan, were sequenced. The genome sizes of NIES-3787, NIES-3804, NIES-3806, and NIES-3807 were 4,524,637, 4,522,701, 4,370,004, and 4,378,226 bp, respectively.

## ANNOUNCEMENT

Cyanobacterial blooms occur widely in freshwater environments worldwide (1). Microcystis aeruginosa is the most well-known bloom-forming cyanobacterium, and it is distributed in eutrophic freshwater environments. The most serious problem associated with this species is the production of hepatotoxic cyanotoxins called microcystins (2, 3). *M. aeruginosa* isolates are genetically divided into at least 12 phylogenetic groups (groups A to K and X) based on multilocus phylogenetic analyses (2, 3). The strains in groups A and X, as well as some B strains, produce microcystins (3, 4). In the current study, we sequenced *M. aeruginosa* strains NIES-3787, NIES-3804, NIES-3806, and NIES-3807, isolated from Lake Kasumigaura, Japan.

Axenic cultures of *M. aeruginosa* NIES-3787, NIES-3804, NIES-3806, and NIES-3807 were obtained from the microbial culture collection of the National Institute for Environmental Studies (https://mcc.nies.go.jp/index.html). These strains were established by using a micropipette under an inverted microscope. The strains were cultured in 10 ml of *Microcystis aeruginosa* medium at 22°C under light at 25 μmol photons m^−2^ s^−1^ with a 12:12-h light/dark cycle. Genomic DNA was extracted from 10-ml cultures of these strains using Agencourt Chloropure (Beckman Coulter) following the manufacturer’s protocol. The resultant DNAs were fragmented to approximately 550 bp using an M220 ultrasonicator (Covaris). Genomic libraries of paired-end reads were constructed using a NEBNext Ultra II DNA library prep kit for Illumina (New England Biolabs). Next-generation sequencing was performed with the MiSeq platform (Illumina) using a 500-cycle MiSeq reagent kit version 2. The resultant paired-end reads for NIES-3787, NIES-3804, NIES-3806, and NIES-3807 were 151,461,029 bp, 643,439,906 bp, 395,828,445 bp, and 197,435,680 bp, respectively. The raw reads were trimmed using Trimmomatic version 0.38 (5), and then *de novo* assembly was performed using SPAdes version 3.11.1 (6) in Shovill version 1.0.4 (https://github.com/tseemann/shovill). Next, the assembled scaffolds were polished using Pilon version 1.22 (7). After the removal of short reads (<200 bp), functional annotation was performed using the DFAST legacy server (8) with CyanoBase (9) as a database. We used CheckM version 1.0.11 to estimate genome completeness (10). Default parameters were used for all software. Group identification analysis of each strain was carried out based on *ftsZ*, one of seven multilocus sequence typing loci (2, 3).

The genome assembly results are detailed in Table 1. As the result of group identification analysis, NIES-3787, NIES-3806, and NIES-3807 were identified as group G, and NIES-3804 was not assigned to any known group. These four strains did not possess a microcystin biosynthetic gene cluster (11). However, some secondary metabolite gene clusters, including aeruginosin (NIES-3787, NIES-3806, and NIES-3807) (12), anabaenopeptin (NIES-3806) (13), microcyclamide (NIES-3804) (14), and micropeptin (NIES-3787 and NIES-3806) (15), were predicted using antiSMASH version 5.0.0 (16). Additional genomic information about *M. aeruginosa* would be useful for monitoring algal blooms and managing freshwater ecosystems.

**TABLE 1:** Characteristics and accession numbers of four Microcystis aeruginosa genomes

Strain name	Assembly size (bp)	No. of contigs	*N*_50_ (bp)	Genome completeness (%)	CheckM contamination (%)	GC content (%)	No. of coding sequences	Accession no. of whole-genome shotgun submissions	SRA accession no.	GenBank assembly accession no.
NIES-3787	4,378,226	214	73,037	99.89	0.66	43.0	4,126	BJCH01000001–BJCH01000214	DRR205020	GCA_009811815
NIES-3804	4,524,637	238	45,562	99.89	0.37	43.0	4,226	BJCI01000001–BJCI01000238	DRR205021	GCA_009811835
NIES-3806	4,522,702	235	67,327	99.89	0.37	43.0	4,180	BJCJ01000001–BJCJ01000235	DRR205022	GCA_009811855
NIES-3807	4,370,004	214	46,356	99.89	0.95	43.0	4,066	BJCK01000001–BJCK01000228	DRR205023	GCA_009811875

### Data availability.

The draft genomic sequences of Microcystis aeruginosa NIES-3787, NIES-3804, NIES-3806, and NIES-3807 have been deposited in DDBJ/EMBL/GenBank under the accession numbers BJCH01000001 to BJCH01000214, BJCI01000001 to BJCI01000238, BJCJ01000001 to BJCJ01000235, and BJCK01000001 to BJCK01000228, respectively. The raw genomic reads of the strains are available in DDBJ/EMBL/GenBank under the accession numbers DRR205020, DRR205021, DRR205022, and DRR205023, respectively.
